# Supplementary material for: Are carbon emissions trading and green financial instruments synergistic? -Comprehensive quantitative research based on content analysis
Source: PLoS One. 2024 Mar 7;19(3):e0298601. doi: 10.1371/journal.pone.0298601 (PMC10919601; doi:10.1371/journal.pone.0298601)
Supplement: S1 Data — (ZIP) [file pone.0298601.s001.zip › Data/地级市Z_F工作报告.zip!/╔╜╢1⁄2/╠⌐░▓╩╨/└┤╫╘╣1⁄2╓┌║┼í╛┬φ┐╦╩2╛▌═°í┐_2009.docx]

各位代表：

现在，我代表市人民政府向大会作工作报告，请予审议，并请市政协委员和其他列席人员提出意见。

一、2008年工作回顾

2008年是极不寻常、极不平凡的一年。一年来，全市人民在中共泰安市委的正确领导下，认真贯彻落实科学发展观，紧紧围绕市委确定的“一三四五”的发展思路，深入开展“继续解放思想推进科学发展”学习教育活动，努力克服各种困难和不利因素影响，坚定信心，攻坚克难，扎实工作，全市经济社会保持了平稳较快发展的态势，较好地完成了全年的目标任务。

（一）经济保持了较高的增长速度和良好的运行质量。预计全市生产总值增长14%左右；完成地方财政收入76.4亿元，增长19.0%，其中各项税收占财政收入的76.7%，同比提高3.9个百分点；规模以上工业企业实现主营业务收入2450亿元、利润170亿元，同比分别增长30%、35%；实现社会消费品零售总额466.6亿元，增长23.3%；全市金融机构人民币各项存、贷款余额达到974.1亿元、605.1亿元，增长19.9%和15.0%。

（二）大项目建设取得新成绩。全市规模以上固定资产投资完成802亿元，增长25.6%。确定的103个投资亿元以上的重点建设项目完成投资296.7亿元，占年度计划投资的108.2%。市和县市区都策划新上了一批符合国家产业政策、促进结构调整、能持续提供税源、有利于增加就业的好项目。如泰山玻纤新材料工业园一期、泰开二期工程、依诺威精梳、泰山轮胎全钢巨型工程胎、山东恒信120万吨焦化和10万吨苯加氢、新矿集团和波兰柯派克斯集团创业园、山东乾元不锈钢极薄板、国华时代五星级大酒店、哈博太阳能光伏产业基地、鼎鑫冷却器等项目，投资规模都比较大，预期效益都比较好。

(三)经济结构调整取得积极进展。农业和农村经济保持良好发展势头。粮食单产、总产均创历史最高水平；新增高效经济作物15万亩、高值田10.3万亩、设施农业5万亩；新增绿色食品认证面积11.5万亩、无公害农产品面积12万亩、有机蔬菜面积4万亩。工业结构进一步优化，主导产业、骨干企业与利税大户培植取得新成绩。规模以上工业实现增加值780亿元，同比增长16.3%；主营业务收入过亿元、10亿元的工业企业分别达到435家、35家，比上年增加77家、11家；利税过千万元、亿元的企业分别达到480家、45家，比上年增加90家、8家。旅游服务业发展明显加快。国内旅游人数1848.7万人次，实现国内旅游收入136.9亿元，分别增长23.0%和30.6%，其中泰山景区进山进景点游客突破300万人、购票进山游客突破210万人。

（四）自主创新、节能减排取得明显成效。全市实现高新技术产业产值占规模以上工业总产值的比重达到28.88%，58项科技成果获国家、省级科学技术奖励，省级以上高新技术企业研发机构达到66个，省以上高新技术企业达到176家，其中国家级23家，泰安跻身“中国城市综合创新能力50强”。万元GDP能耗在上年降低4.7%的基础上又实现了更大幅度的下降。创建省级节水型城市通过考核验收。

（五）改革开放继续深化。国有资产出资人制度逐步完善，国资监管运营质量明显提高；国有企业改革改制后续工作和行政事业单位国有资产管理体制改革稳妥推进。投融资体制改革更加深化和完善，银企合作、企业上市、争取地方金融机构试点、异地银行来泰放贷等取得积极进展。事业单位改革、体制调整和机构编制资源整合工作迈出新步伐，节能减排统计、困难职工帮扶、社情民意调查、安全生产应急救援等工作机构得到加强，组建了动物疫病预防控制和动物卫生监督机构。农村土地流转、林权、乡镇综合配套等改革有效实施。对外开放的质量和水平逐步提高，对外区域合作与交流日趋活跃。全市新批利用外资项目118个，实际利用外资11.3亿美元，增长15%；新开工国内招商引资总投资3000万元以上的项目520个，实际到位资金207亿元，增长18%；全市外贸进出口总值完成15.8亿美元，增长18.5%，其中出口9.5亿美元，增长12%；签订对外承包劳务合同额5.8亿美元，完成营业额4.1亿美元，外派劳务18667人，分别增长27.0%、29.5%和28.2%。

（六）城镇规划建设管理得到加强。严格执行新一轮城市总体规划，组织编制了泰安历史文化名城保护、泰山环山带状公园、蒿里山历史文化区保护、绿地系统、商业网点、综合交通等规划；制定了时代发展线、长城路、泮河绿化风光带、财源商业街等详细规划。大力实施城市建设重点工程，泰城重点完成了环山路东段、迎春西路、学院路、万官路等建设工程和七里河、唐庄河、泮河大街等污水治理工程，长城路区片、城中村改造等规划建设加快推进。全力开展城乡环境综合整治，集中对泰城经营、治安、交通、建设、旅游等秩序进行了综合治理，环境面貌得到明显改善。县市驻地和小城镇规划、建设与管理取得新进展。市高新区以建成“经济高速增长区、高新技术聚集区、集约高效样板区、科学发展示范区”为目标，经济发展、项目建设、招商引资等取得新成绩。泰山景区保护管理不断强化，景区基础设施和环境秩序进一步改善。

（七）和谐社会建设稳步推进。城镇居民人均可支配收入达到16000元、农民人均纯收入达到6193元，同比分别增长15.8%和14.5%。基础教育得到加强，普通高中、职业教育、高等教育都有新的发展；城乡义务教育经费保障机制逐步完善，全市义务教育段共有45.9万名学生享受到免费政策。公共文化服务体系逐步完善，四大文化“惠民工程”积极推进，全市有10件作品获省精品工程奖。城乡公共卫生服务、预防保健、疾病控制、医疗救治得到进一步加强。低生育水平保持稳定，在全省综合考核中继续保持先进位次。全市新增城镇就业再就业7.2万人，城镇登记失业率控制在3.1%。全市共收缴各项社会保险费39.6亿元，发放29.6亿元；城镇居民基本医疗保险制度全面建立，失业保险、城乡低保、五保供养、新型农村合作医疗补助标准得到提高。全市建设经济适用住房19.8万平方米、廉租住房1.5万平方米，实施了农村贫困残疾人危房改造工程；全市累计归集住房公积金31.3亿元，为2.6万名职工发放贷款22.6亿元。全市行政村通油路率、通客车率分别达到96.3%、99.5%。完成农村无害化生态卫生厕所6万座，新建户用沼气池1.6万个。“平安泰安”建设取得新的进展，严打整治斗争深入推进，治安防控体系不断完善。奥运火炬在我市安全顺利传递，奥运金牌实现零的突破。抗震救灾和援建工作取得阶段性重要成果。精神文明建设深入开展，泰城获得省级文明城市称号，泰山通过全国文明风景旅游区复审，莲花山被评为“国家4A级景区”。民主法制得到加强，国防动员、民兵预备役、双拥共建取得新成绩。广播电视、新闻出版、外事侨务、宗教、气象、地震、档案、史志、老龄、残疾人等项社会事业全面进步。

（八）政府行政服务水平进一步提高。认真贯彻实施《监察法》、《行政监察法》，在全市行政执法部门实行了首次不罚教育警示制度和行政处罚备案制度，建立了行政执法资格监督备案制度和货物运输“绿色通道”制度。深入实施《公务员法》，大力加强公务员队伍建设，加大公务员培训力度，干部队伍素质和施政能力不断提高。坚持依法行政，规范执法行为，优化执法环境。加强行政审批服务标准化和网络审批服务平台建设，规范服务行为，强化协调监督，审批服务质量和效率进一步提高。政务信息化建设扎实推进，政务公开、便民服务、行风评议等更加深入开展。加快推进政府职能转变，政府服务效率和质量有了明显提高。

各位代表，在历史罕见的挑战和风险面前，全市经济社会发展取得这样的成绩是十分不容易的，这是我们在市委正确领导下，认真贯彻中央和省决策部署、全面落实科学发展观的结果；是全市人民继续解放思想、凝心聚力、艰苦奋斗的结果；也是驻泰各单位和各界人士鼎力支持、自觉融入泰安经济与社会发展的结果。在此，我代表泰安市人民政府，向全市广大干部群众、各民主党派、工商联、无党派爱国人士，向人民解放军和武警驻泰部队及省以上驻泰单位，向关心支持泰安发展的港澳台同胞、海外侨胞及海内外朋友，表示崇高的敬意和衷心的感谢！

在充分肯定成绩的同时，也要清醒地看到我们面临的严峻形势和发展中存在的困难和问题。由美国次贷危机引发的金融危机已从局部发展到全球，从发达国家传导到新兴市场国家和发展中国家，从金融领域扩展到实体经济领域。当前我市经济社会发展中存在的主要问题，一是受国内外各种不确定因素相互叠加影响，经济增长速度总体放缓、效益下滑；市场需求萎缩，企业生产经营、外贸出口、招商引资遇到前所未有的困难。二是财政增幅回落，国家实行结构性减税措施，清理和减免行政性收费，政策性减收因素增多，抗震救灾、支持三农、社会保障等刚性增支因素增多，保财政收支平衡任务艰巨。三是受经济放缓、企业经营困难影响，就业再就业压力很大；农产品价格下行和农民工回流，农民持续增收难度加大。四是经济结构性矛盾依然突出，偏重型的工业结构给节能减排带来很大压力。五是领导驾驭科学发展的水平还需进一步提高，争先进位的意识需不断增强，一些重点工作推进力度需逐步加大，关注和改善民生、提高行政效能、优化发展环境等还有大量的工作要做。对此，我们必须充分认识形势的严峻性和复杂性，增强忧患意识，保持清醒头脑，把困难估计得更充分一些，把措施准备得更周密一些，以更加高昂的斗志和奋发有为的姿态，积极主动地应对各种困难挑战，确保全市经济与社会平稳较快发展。

二、2009年工作目标要求

2009年是建国60周年，也是推进“十一五”规划顺利实施的关键一年。政府工作的总体要求是：全面贯彻党的十七大、十七届三中全会和中央、全省经济工作会议精神，坚持以邓小平理论和“三个代表”重要思想为指导，深入落实科学发展观，按照市委确定的“一三四五”发展思路，以项目建设为抓手，以改革创新为动力，积极应对危机挑战，转变发展方式，调整经济结构，扩大内在需求，促进经济增长，努力改善民生，调动一切积极因素和力量，坚定信心，迎难而上，确保全市经济平稳较快发展，推进社会全面进步，加快“建设经济文化强市、打造国际旅游名城”步伐。主要预期目标和约束性指标是:全市生产总值增长12%；万元GDP能耗、取水量分别下降5.94%和6%；二氧化硫和COD排放量分别下降2.5%和0.5%；地方财政收入增长10%；全社会固定资产投资增长22%；社会消费品零售额增长16%；外贸进出口总值增长12%；城镇居民人均可支配收入和农民人均纯收入均增长8%；新增城镇就业再就业5.7万人，城镇登记失业率控制在3.8%以内；人口自然增长率控制在5.6‰以内。在实际工作中力求发展得更好一些、更快一些。

做好今年的工作，挑战和机遇并存。我们既要看到金融危机带来的严峻挑战，更要注重在逆境中发现和培育积极因素，从变化的形势中捕捉和把握难得的发展机遇。要看到国际金融危机对原有经济发展格局和模式产生巨大冲击，这是我们提升产业层次、提高核心竞争力、在新一轮竞争中实现跨越发展的重大机遇。国家实施扩大投资、保经济增长的战略举措，为我们改善基础设施、调整经济结构、淘汰落后产能提供了难得的良好机遇。省委、省政府“一体两翼”区域发展战略的实施，我们既可接受“济南都市圈”、胶东半岛城市群的辐射，又享受鲁南经济带的优惠政策，两相受益，为我们区域经济发展提供了更为广阔的空间。经过30年的改革开放，我市的综合实力显著增强，保经济平稳较快发展有着稳固的基础和条件，尤其是近几年我们坚持不懈地抓大项目建设，发展的后劲逐步增强，随着时间的推移其效果将逐步凸显。面对困难，信心是金。只要我们审时度势，坚定信心，抢抓机遇，积极作为，就一定能够促进全市经济与社会平稳较快发展。在工作指导和把握上，要做到“五个坚持”：坚持以科学发展观统揽全局，紧紧扭住发展第一要务不放松，把握发展规律、创新发展观念、破解发展难题，统筹兼顾，整体推进经济与社会全面协调可持续发展；坚持继续解放思想，牢固树立“四种意识”、推进“四个创新”、实现“四个提高”，以思想的大解放、观念的大更新推进经济社会大发展；坚持把上级的决策部署与泰安实际相结合，按照市委确定的工作思路，把保经济增长作为重中之重，把发展方式转变和结构调整作为主攻方向，把大项目建设作为基本抓手，聚精会神扩内需，一心一意保增长；坚持把深化改革、扩大开放作为推进经济发展的强大动力，积极推进重点领域和关键环节的改革，努力提高对外开放水平和抵御外部经济风险的能力；坚持以人为本，更加注重改善民生，大力实施全民创业战略，着力解决涉及群众利益的热点难点问题，让更多的群众共享发展成果，凝聚全社会的力量，形成保经济与社会平稳较快发展的强大合力。

三、2009年工作重点

推进今年全市经济与社会平稳较快发展，必须统筹谋划，重点突破，围绕“保增长、保民生、保稳定，调结构、调布局、调产品，扩内需、扩市场、扩总量”的要求，紧紧抓住牵动全局的重点工作、事关群众利益的突出问题，强力推进。

（一）坚持不懈地抓好大项目建设，努力扩大投资规模

项目建设特别是大项目建设，是培植财源、扩大就业、实现科学发展的基本抓手，是提升一个地区经济综合实力的重要支撑，也是拉动内需、扩大投资的重要载体。按照“投产一批、开工一批、储备一批”的要求，集中力量抓好泰山玻纤六条8万吨生产线、泰山区西变超高变压器、岱岳区科诺型钢、新泰市多晶硅和碳纤维新材料、肥城市石墨电极和甲酸四期扩能、宁阳县黄河电缆、东平县中顺集团高档系列生活用纸等一批投资亿元以上的续建和新开工项目建设，使其尽早形成新的经济增长点。紧紧围绕结构调整、经济发展方式转变、提升城市综合竞争力，依托高层次专家咨询机构，论证、策划、储备一批投资亿元以上符合国家产业政策、科技含量高、能持续提供税源的大项目、好项目。在遵守国家最严格耕地保护制度和节约集约用地制度的前提下，集中土地资源保重点项目建设。创新完善大项目建设推进机制，进一步强化重大项目领导包保责任制，对列入市重点的项目，实行集中调度和专项督查制度，提高行政服务效率和质量，确保项目早开工建设、早见到效益。

扩大投资规模，是扩大内需、确保增长的重要途径。必须坚定不移地加大投资力度，优化投资结构，充分发挥投资对经济的拉动作用。紧紧抓住中央扩大预算内投资的重大机遇，切实抓好已对接落实项目的建设。紧紧围绕农业和农村基础设施、铁路公路等重大基础设施、企业技术改造、旅游服务业、自主创新和节能减排等，继续筛选一批符合中央投资方向、开工条件成熟、有利于结构调整、带动能力强的项目，及时上报对接，力争有更多的项目进入国家和省的投资盘子，努力在中央和省预算新增投资中争取更多份额。紧紧抓住国家实行积极财政政策和适度宽松货币政策的机遇，切实加强财税金融工作，充分发挥财税金融对经济发展的支持作用，通过招商引资、争取上级投资、加强银企联合、推进企业上市、吸引社会资本等手段，培育新的投资主体，拓宽投融资渠道，努力扩大投资规模。优化投资结构，严格控制“两高一资”和产能过剩行业投资，防止低水平重复建设和盲目扩张。

（二）加快发展特色现代农业，积极推进社会主义新农村建设

坚持把发展农村经济、提高农民收入作为扩大内需的重要内容，进一步强化农业基础地位。全面实施优质粮食高产创建工程，加强粮食调控管理，确保粮食和主要农产品有效供给。大力实施“一村一品百村示范千村推进工程”，加快发展有机蔬菜、苗木花卉等优质、高效、安全、特色农业，积极推进“奶业富民工程”。年内力争新调整高效经济作物15万亩，新增有机食品认证面积5万亩，其中有机蔬菜3万亩。加快构建现代农业产业体系，突出抓好农产品加工龙头企业、农民专业合作社发展和农村市场体系建设，提升农业产业化水平。年内引进新建规模以上农产品加工龙头企业35家，新建农民专业合作社100个。加快农业科技进步，大力实施农业良种产业化、科技特派员、农村科技信息村村通、名特优果品科技示范工程，建立多元化农业科技推广体系。加快农产品质量检测体系和出口标准化基地建设，确保农产品质量安全。实施“百库千塘”治理工程，抓好水土流失综合治理、节水灌溉、植树造林和农业综合开发、基本农田建设。加大公共财政对农村的投入力度，继续抓好农村“路水电医学”、养老院等基础设施和公共事业建设。科学规划，分步实施，有计划地搞好农村新型社区建设。大力发展沼气、太阳能等再生清洁能源，加大农村改厕力度，加强农村环境综合整治。高度重视农民工就业和农民增收工作，采取加大就业培训、政策优惠、小额贷款等方式，引导农村劳动力就地就业创业，鼓励返乡农民工创业，多渠道转移农村劳动力，最大限度地拓展农村劳动就业渠道和农村内部增收空间。全市新增农村劳动力转移就业6万人。认真落实支农惠农强农政策，充分调动广大农民搞好生产经营、增加收入的积极性和创造性。加强农村基层组织建设，健全民主管理制度，增强农村干部发展经济、维护稳定、带领农民致富的能力。

（三）继续做强做大工业经济，努力增强综合竞争力和抗御风险的能力

坚持走新型工业化道路不动摇，大力推进信息化与工业化的融合，逐步形成主导产业突出、骨干企业众多、创新能力和核心竞争力较强的工业体系。面对国内外复杂困难的经济环境，切实加强对工业生产的运行调度、监测分析和组织协调，帮助解决重要生产要素供求衔接中存在的突出问题，特别是加强对30户重点企业生产销售情况的监测分析，引导企业全力开拓国内外市场，加强企业内部管理，帮助骨干企业度过难关，发展壮大。紧紧抓住国家扩大内需的重大机遇，通过膨胀规模、产业升级、链条延伸、市场开拓等途径，进一步加大输变电设备、汽车及零部件、无机非金属新材料、精细化工等主导产业和骨干企业培植力度，加快泰开工业园、玻纤工业园、中国重汽工业园、羊流起重机械园区、信发集团盐化工园、华丰工业园、瑞星工业园等重点园区建设，抓好一批重点企业百亿扩规项目实施，不断提高工业经济的竞争力。高度重视高新技术产业发展，大力发展电子信息、新材料、光机电一体化、新能源和高效节能高新技术产业，筹划发展光伏产业。充分用好增值税转型、进口设备免税等优惠政策，大力推进企业技术改造，全年企业技术改造投入要占到工业投入的40%以上。以国家新颁布的九大支柱产业振兴规划为指导，鼓励行业龙头企业、优势企业兼并重组落后企业和困难企业，鼓励优势企业强强联合，鼓励关联产业、上下游企业联合重组，提高产业集中度和抗风险能力。要坚持“抓大”与“促小”并举，大力实施全民创业战略，健全完善中小企业担保体系，加大对中小企业的政策和金融扶持力度，培育一大批成长性强、对财税贡献大的中小企业和特色产业集群。

（四）加快发展以旅游为龙头的服务业，努力提升档次和对经济增长的贡献率

坚持把发展旅游服务业，作为扩大最终消费、带动中间需求、保持经济平稳较快增长的重要途径。一是努力推进旅游业实现新突破。围绕“吸引人、留住人”，创新体制机制，整合旅游资源，在推动泰山、泰城为中心旅游项目建设的同时，积极推进徂徕山、莲花山、东平湖等旅游资源开发，形成大泰山旅游格局。实施项目带动战略，策划建设一批体量大、带动力强、能够整合旅游各要素的休闲度假、康体娱乐、农业观光和特色高端旅游项目，重点抓好泰山方特欢乐世界、环山路旅游观光线、泰山实景演出、艾洼生态民俗村、金斗梨苑休闲广场、东平湖旅游航道和旅游码头服务区等项目建设，变单一观光型旅游为观光休闲度假型旅游。高度重视抓好旅游市场主体培育，引进、培强、做大一批旅游骨干企业。着力抓好高档星级酒店、特色餐饮美食街、旅游购物休闲街、旅游集散中心建设，完善城市停车场、景区和泰城旅游标识，完成泰山连接徂徕山快速通道建设，加强旅游纪念品开发生产，提高旅游产业各要素服务水平。进一步加大旅游宣传促销力度，努力增强泰山、泰安的美誉度和吸引力。二是高度重视服务业特别是现代服务业发展。认真落实促进服务业发展的各项政策，鼓励、支持、引导各类社会资本进入服务业领域。加快发展现代物流、科技信息、金融保险、中介服务等现代服务业。加快推进服务业载体建设，积极推进“1030”工程，重点抓好一批服务业集聚区和大型商贸流通企业和品牌。大力开拓农村市场，扩大农村消费，着力抓好万村千乡市场工程和家电下乡工作。三是高度重视房地产业发展。认真贯彻国家关于房地产业健康发展的政策，按照省委、省政府关于“保住一头、启动一头、放开一头”的要求，落实好政府资金、土地供应和各项优惠政策，加快实施保障性安居工程建设；研究制定货币支持与实物支持相结合的办法，大力启动农村住房消费；对中高档商品房落实放宽二套房政策，鼓励普通商品房消费。四是大力发展文化事业和文化产业。实施文化建设“八大工程”，积极推进文化体制改革，继续抓好“乡村阅读工程”、“农家书屋”、乡镇综合文化站等文化设施建设，健全覆盖全社会的公共文化服务体系。开展对泰山文化的深度挖掘和研究，建设一批科技含量高、附加值高的文化产业项目，加快形成以泰山文化为品牌的泰山文化产业聚集区，提高文化产业对经济增长的贡献率，形成新的增长点。

（五）加大自主创新、节能减排力度，增强经济竞争力和可持续发展能力

创新是经济社会发展的不竭动力和永恒的主题。加快创新型泰安建设，继续坚持做好“自主知识产权、消化吸收再创新、科技成果转化”三篇文章，使自主创新成为产业结构调整、新农村建设、和谐社会建设的有力支持。积极推进企业观念创新、制度创新、技术创新和管理创新，全面提升企业核心竞争力。大力实施知识产权战略，积极争创国家知识产权示范城市。积极争创省双百重点技术中心、省级技术中心和省级工程技术研究中心，抓好30家创新型企业、30个驰名商标或著名商标、30个省以上名牌的培植，年内完成1000项技术创新项目。加快建立以企业为主体、市场为导向、产学研相结合的技术创新体系，鼓励企业采取多种形式加强与国内外知名企业、科研院所的技术和资金合作，引导和支持创新要素向企业集聚，促进科技成果向现实生产力转化。大力实施人才强市战略，统筹抓好企业经营管理人才、专业技术人才、高技能人才、农村实用人才队伍建设，完善人才培养、选拔、激励机制，在全社会形成良好的创新创业环境，尤其是要高度重视抓好企业家队伍建设，培养一批具有战略性眼光、战略性思维与胆识、面对复杂多变形势能够实施战略性举措的企业家。

节能减排是推进发展方式转变、实现可持续发展的重大战略举措。坚持节约资源和保护环境基本国策，加大依法治理力度，强化企业社会责任，坚定不移地打好节能减排攻坚战，确保完成各项节能减排约束性指标。认真落实国家节能减排产业政策，强化源头控制，加快淘汰落后产能，抓好九大高耗能行业和百户重点企业的节能减排管理。积极推进节能技术进步和节能项目建设，大力发展循环经济，继续实施好“1623培育计划”，着力抓好清洁生产、资源综合利用和可再生资源回收利用，节约集约利用土地、水和矿产、河砂等资源。大力实施主要污染物总量削减、全运会空气质量保障、南水北调水污染防治三项工程。积极实施《泰安生态市建设总体规划》，努力建立经济发展与资源节约、环境保护、生态建设相互促进的发展机制，改善城乡人居环境，确保生态环境安全。

（六）坚定不移地推进改革开放，增强平稳较快发展的动力和活力

化解和应对国际金融危机，做好今年的政府工作，必须加大改革的力度，着力构建充满活力、富有效率、更加开放、有利于科学发展的体制机制。继续深化国有企业改革，推进企业公司制改造，完善法人治理结构，健全现代企业制度。进一步完善国有资产管理制度，提高国有资本运营效益。深化行政事业单位国有资产管理体制改革。积极推进财税体制改革，全面实施增值税转型和燃油税费改革，清理规范行政性收费。积极推进投融资体制改革，规范政府投资管理，探索多元融资方式和途径。培育壮大劳动力、土地、技术、资本等要素市场，规范发展市场中介组织，加快建设统一开放、竞争有序的现代市场体系。深化收入分配、事业单位人事制度和事业单位分类改革。坚持农村土地承包关系保持稳定并长久不变，在不改变土地集体所有性质和用途的前提下，允许农民按照依法自愿有偿的原则流转土地承包经营权，继续深化农村金融、集体林权和农村综合配套改革。

进一步扩大对外开放，更好地利用国际国内两个市场、两种资源，拓展对外开放的广度和深度。坚持内外并举，千方百计扩大招商引资。充分利用产业、资源、环境、服务等要素，发挥企业招商的主体作用和园区、项目的载体作用，创新利用外资方式，围绕建设经济文化强市、打造国际旅游名城，在培植壮大工业主导产业、发展旅游服务业和现代农业上做好招商引资的文章，在引进国内外大企业大集团上下功夫，努力扩大招商引资规模，提高招商引资的质量和效益。充分利用国际金融危机的机遇，加快引进国内外的先进技术、关键设备和重要能源原材料，促进企业进行合资合作、股权并购和兼并重组。努力扩大对外区域合作与交流。按照省委提出的“一体两翼”战略部署，积极融入“济南都市圈”、胶东半岛城市群，用足用好鲁南经济带优惠政策，着力推动列入“一体”规划中的23个项目建设。加强与环渤海经济圈、淮海经济协作区、滨海新区经济技术的联系融合。更加积极稳妥地实施“走出去”战略，鼓励引导有实力、有信誉的企业到国外投资办企业、承揽工程与开展劳务合作。坚持走以质取胜、集约化、多元化的发展路子，转变对外贸易增长方式，加大国际市场开拓力度，大力培植出口骨干企业和利税大户，努力扩大具有自主品牌和高附加值产品出口，增加服务产品和农产品出口。

（七）加强城镇规划建设管理，增强城镇对经济的承载力和辐射作用

坚持连片开发、精品化、精细化原则，高起点规划、高标准建设、高水平管理，创新城市发展理念，突出城市发展特色，加快推进城市化进程。进一步强化规划的刚性龙头作用，严格按照新一轮城市总体规划、各专项规划和控制性详细规划进行建设，重点抓好两线（时代发展线、历史文化轴线）、两带（沿山景观带、泮河景观带）和长城路沿线及京沪高铁车站组团等重点工程，努力提升泰城的综合服务功能，改善人居创业环境。切实加强城市精细化管理，巩固泰城综合整治成果，进一步加大城区道路改造、污水治理、市政公用设施建设和城中村改造、旧小区整治力度，努力提升城市的品位、质量和档次。充分发挥城市发展投融资管理中心的职能作用，整合、开发、利用各类城市资源，拓宽城市建设融资渠道。进一步加大县市驻地城市和小城镇规划建设力度，积极推进城市规划建设管理向城乡结合部和小城镇延伸，提升城镇的公共服务功能和对各类要素的承载能力。加快各类园区建设，市高新区按照市委提出的“四区”目标要求，突出项目引进、基础设施建设、体制创新、自主创新、节能减排和改善民生等重点，进一步加大开发建设力度，增强经济集聚集约发展能力和带动辐射作用。

（八）更加高度关注和改善民生，加快发展各项社会事业

把改善民生作为促进平稳较快发展的出发点和落脚点，集中财力，重点办好一批经济发展急需、人民群众热切期盼的实事。实施更加积极的就业政策，通过发展经济创造就业岗位、自主创业、积极开发公益性就业岗位、劳务输出等措施，全方位促进就业增长，着力解决好“4050”人员、“双零”家庭、被征地农民、城镇失业人员等困难群体就业再就业问题。高度重视大学毕业生就业，实施大学生科技创业助推计划，抓好“三支一扶”、“一村一名大学生”工作，切实做好军转干部、退役士兵安置工作。加快健全完善以养老、医疗、失业保险为重点的城乡社会保障体系，加大社会保险扩面征缴力度，提高保障能力。扩大城镇居民基本医疗保险覆盖面，提高企业离退休人员养老金、城乡低保水平和优抚对象优待标准。企业退休人员养老金月人均增加125元，农村低保标准由最低每年每人900元提高到1000元以上。大力实施住房保障安居工程，抓好经济适用房、廉租住房建设和农村危房改造试点，全市新增廉租住房8.1万平方米。进一步加大住房公积金征收、信贷和监管力度，确保安全有效运作。加大对特困户、灾民等困难群体的救助，大力发展社会福利和慈善事业。进一步加大“平安泰安”建设力度，完善社会治安防控体系，深入推进严打整治斗争，着力解决涉及民生的治安热点、难点问题。切实做好安全生产工作，做到安全警钟长鸣、安全隐患常排、安全事故严查，重点加强矿山、化工、交通、病险水库等重点行业、重点企业、重点部位的安全管理，坚决遏制重特大安全事故。高度重视并切实抓好食品药品质量安全，确保人民群众生命健康。加强和完善社会管理，健全应急管理体制机制，高度重视人民群众来信来访，进一步健全社会矛盾纠纷调处机制，着力解决群众反映强烈的突出问题，及时化解各种社会矛盾，全力维护社会稳定。

加快繁荣发展各项社会事业。坚持教育优先发展战略，完善义务教育经费投入保障机制，加强各阶段学生素质教育，大力发展与产业结构调整和就业需求相适应的职业教育特别是农村职业教育，继续大力支持驻泰高校的改革与发展。积极推进医药卫生体制改革，提高公共卫生事件应急处置能力，搞好重大传染疾病防控；巩固提高新型农村合作医疗建设成果，加大乡镇卫生院改造，加快提升农村卫生室和城市社区卫生服务水平，财政对参加新型农村合作医疗农民的补助提高到每人每年80元。大力开展爱国卫生运动。加强体育基础设施建设，深入开展全民健身活动，积极组织协办好第十一届全运会。依法加强宗教事务管理，认真落实少数民族政策，大力发展少数民族经济。继续加强人口和计划生育工作，稳定低生育水平，提高出生人口素质。加强民主法制建设，完善基层民主管理制度。加快社会主义核心价值体系建设，大力推进群众性精神文明创建活动、公民道德建设、青少年思想道德建设。重视抓好国防动员、民兵预备役、双拥共建工作。关心支持妇女儿童、残疾人事业，做好外事、侨务和档案、气象、防震、减灾、救灾等各项工作。

四、政府自身建设

困难严峻的形势和艰巨繁重的任务，对政府工作提出了新的更高要求。各级政府必须进一步解放思想，更新观念，转变职能，提升素质，增强驾驭科学发展和应对困难局面的能力。

要科学务实。全体政府工作人员必须进一步加强对科学发展观理论的学习，深刻领会、准确把握其本质内涵，增强落实科学发展观的自觉性和坚定性，牢牢扭住发展第一要务不放松，坚持以人为本、统筹兼顾，推动经济与社会协调可持续发展。坚持以科学的方法推进工作，准确把握发展规律，科学谋划发展思路，创新转变发展模式，有效破解发展难题。密切关注国际国内经济形势的变化，把上级决策部署与泰安实际结合起来，顺势而为，积极作为，奋发有为。健全完善科学发展目标体系、责任落实和奖惩考核机制，形成正确的导向机制、客观的评价机制、有效的激励机制和严格的约束机制。坚持求实务实，狠抓落实，克服官僚主义、形式主义，力戒空谈，崇尚实干，不断取得新成效。

要开拓创新。全体政府工作人员，要进一步提升发展境界，把应对金融危机、保持经济增长的过程，变成调整经济结构、转变发展方式的过程，变成增强自主创新能力、促进节能减排的过程，变成改善民生、促进社会和谐的过程。要充分发挥市场配置资源的基础性作用，实施行政强力推动与市场化相结合，创造性地开展工作。要大力推进政府管理创新，整合集约利用行政服务资源，推进政务服务信息化，降低客商和市民获取政府服务的成本。要进一步振奋精神，敢为人先、敢于争先，知难而进、迎难而上，始终保持干事创业、顽强拼搏的高昂斗志，坚定战胜困难、促进平稳发展的信心和决心，激发全社会创新创造创业的活力和热情。

要依法行政。进一步强化法制意识，认真贯彻落实各项法律法规，深入开展“五五”普法和“四五”依法治市，提高全社会自觉学法守法用法的意识，夯实社会和谐的法治基础。大力加强行政执法队伍建设，提高依法施政水平，努力做到行政合法、程序正当、高效便民、诚实守信。进一步健全完善执法责任、效能监察、举报投诉等制度，确保权力依法公正运行，确保权力始终受到监督。坚持向人大报告工作和向政协通报工作制度，认真执行人大及其常委会的决议、决定，自觉接受人大的法律监督和政协的民主监督，认真办理人大代表议案、建议和政协提案。加强同各民主党派、工商联、人民团体和社会各界人士的联系。

要廉勤为民。全体政府工作人员特别是领导同志，要自觉遵守党的纪律和廉洁自律的各项规定，常思贪欲之害，常怀律己之心，常省自身之过，警钟长鸣，防微杜渐。坚持抓源治本，从严治政，认真落实廉政建设责任制，高度重视加强制度建设和监督制约，严格按照法定权限和程序行使权力、履行职责。继续开展好行风评议、行风热线等活动，坚决查办违纪违法案件，重点加强主要领导干部、关键岗位人员和资金使用、工程建设的审计监督。强化责任意识，转变工作作风，加强调查研究，提高行政效率和服务质量。强化“宗旨”观念，带着感情和责任，体察民情民心，关心群众疾苦，真心实意为群众办实事、解难题、谋福祉，切实让广大群众享受到改革与发展的成果，使政府赢得广大人民群众的信赖与支持。

各位代表！困难与挑战阻挡不住我们前进的步伐，时代召唤着我们，人民群众期盼着我们。让我们高举中国特色社会主义伟大旗帜，更加紧密地团结在以胡锦涛同志为总书记的党中央周围，全面贯彻落实科学发展观，在市委的坚强领导下，进一步解放思想，振奋精神，攻坚克难，顽强拼搏，为实现泰安经济与社会又好又快发展、加快推进“建设经济文化强市、打造国际旅游名城”进程而努力奋斗！
